# A double-hurdle model estimation of cocoa farmers’ willingness to pay for crop insurance in Ghana

**DOI:** 10.1186/s40064-016-2561-2

**Published:** 2016-06-24

**Authors:** Elvis Dartey Okoffo, Elisha Kwaku Denkyirah, Derick Taylor Adu, Benedicta Yayra Fosu-Mensah

**Affiliations:** Institute for Environment and Sanitation Studies (IESS), University of Ghana, P. O. Box 209, Legon, Accra, Ghana; Department of Agricultural Economics and Agribusiness, University of Ghana, P. O. Box LG 68, Legon, Accra, Ghana

**Keywords:** Agriculture, Crop insurance, Cocoa, Risk, Willingness to pay, Premium

## Abstract

Agriculture is an important sector in Ghana’s economy, however, with high risk due to natural factors like climate change, pests and diseases and bush fires among others. Farmers in the Brong-Ahafo region of Ghana which is known as one of the major cocoa producing regions, face these risks which sometimes results in crop failure. The need for farmers to therefore insure their farms against crop loss is crucial. Insurance has been a measure to guard against risk. The aim of this study was to assess cocoa farmers’ willingness to access crop insurance, the factors affecting willingness to pay (WTP) for crop insurance scheme and insurance companies’ willingness to provide crop insurance to cocoa farmers. Multi-stage sampling technique was used to sample 240 farmers from four communities in the Dormaa West District in Brong-Ahafo Region. The double-hurdle model shows that age, marital status and education significantly and positively influenced cocoa farmer’s willingness to insure their farms whiles household size and cropped area negatively influenced farmers’ willingness to insure their farms. Similarly, age, household size and cropped area significantly and positively influenced the premium cocoa farmers were willing to pay whiles marital status and cocoa income negatively influenced the premium farmers were willing to pay. The contingent valuation method shows that the maximum, minimum and average amounts cocoa farmers are willing to pay for crop insurance per production cost per acre was GH¢128.40, GH¢32.10 and GH¢49.32 respectively. Insurance companies do not have crop insurance policy but willing to provide crop insurance policy to cocoa farmers on a condition that farmers adopt modern cultivation practices to reduce the level of risk. The study recommends that cocoa farmers should be well educated on crop insurance and should be involved in planning the crop insurance scheme in order to conclude on the premium to be paid by them.

## Background

Agriculture has been the key player in Ghana’s economic growth and development since independence (Mahrizal et al. 2014). It employs about 70 % of the labour force in Ghana, accounts for about 30 % of Ghana’s GDP and contributes about 60 % of foreign exchange earnings through export (Ayisu 2008; ISSER 2010). Cocoa, coffee, oil palm and rubber are the main cash crops and agricultural export commodities in Ghana. Among these crops, cocoa (Theobroma cacao) is the major agricultural export commodity in Ghana (Anim-Kwapong and Frimpong 2004). It is successfully grown in six out of the ten regions of Ghana, namely, Ashanti, Brong-Ahafo, Central, Eastern, Western and Volta (Anim-Kwapong and Frimpong 2004). Ghana is the second largest producer of cocoa in the world after Cote D’Ivoire and is renowned for its premium quality cocoa bean (Ntiamoah and Afrane 2008; Gockowski et al. 2011). The crop contributes substantially to foreign revenue earnings, employment and domestic incomes (Ayenor et al. 2007; Anang 2011). A report by ISSER (2014) revealed that in 2013, approximately 16.48 % (US$ 2267.3 million) of total agriculture export receipts were foreign revenue earnings from cocoa. Appiah (2004), Anim-Kwapong and Frimpong (2004) and Danso-Abbeam et al. (2014) assert that in Ghana, over eight hundred thousand (800,000) smallholder farm families are employed by the cocoa sector which contributes about 70–100 % of their annual household income. In addition, Asamoah and Baah (2003) and Anim-Kwapong and Frimpong (2004) emphasize that other stakeholders like agrochemical companies, input distributors and licensed cocoa buying companies also depend largely on cocoa for markets of their products, employment and income.

In spite of the significant contribution of cocoa production to Ghana’s economy, it is faced with many challenges such as climate change, bush fire, pests and diseases outbreak, among others resulting in crop failure and/or destruction of crops. According to Laux et al. (2010) and IPCC (2014), the spatial and temporal variability of rainfall which is reflected by recurrent dry spells and floods is the most important factor affecting crop productivity, and hence reducing food security. In addition, Johny et al. (2003) and Bailey et al. (2005) have reported that the yield of cocoa is limited by pests and diseases in many producing countries. Lass (2004), Lanaud et al. (2009) and ICCO (2015) reported that 30–40 % of cocoa produced globally is lost to insect pests and diseases. These challenges pose high risk of crop loss as most farmers in Ghana are smallholders with less input and total dependence on rainfall as source of moisture for crop growth. These factors which are sometimes beyond the control of farmers make farming a risky business, hence, farmers have to manage these risks as part of the general management of the farming business. Some farmers over the years have adopted some measures such as crop diversification, increasing pesticide and fertilizer applications and planting of drought resistant crops to reduce the risk of total crop failure. However, risk continues to be a major factor in agriculture, hence, the need for farmers to guard against risk through farm insurance scheme is crucial.

According to Freshwater and Jette-Nantel (2008), there are two predominant risks that affect the income of farmers and agribusinesses. These risks are price risk and production risk. Price risk comprise of the variations in market prices for agricultural commodities and production inputs whiles production risk is the variations in the quantity and quality of agricultural commodities produced. Although there are several sources of production risk, weather is seen as the most pervasive source of production risk and its impact is felt particularly in countries that rely on rain-fed agriculture (Swiss 2007). Weather variations can severely affect the quality and quantity of crop yield and this is mostly felt in developing countries where majority of agricultural activities remain highly susceptible to extreme and uncontrollable weather events such as insufficient rainfall and extreme temperatures. Poor and vulnerable farming households (with majority being subsistence farmers) are mostly affected by risk due to the weather (Aidoo et al. 2014).

A risk management tool in farming could be insurance. Quagrainie (2006) stated that insurance is a tool to reduce the financial risk of adverse events such as loss of life, medical expenses, auto accidents, casualty losses and weather damage. Similarly, Adams (1995) defines insurance as a contract between two parties, where one party called insurer takes a premium from the other party (insuree) in order to pay a fixed amount of money to the insuree when there is occurrence of an unforeseen event. Insurance provides the opportunity for people to replace risk with known cost. People purchase insurance coverage with the promise of receiving an amount from the insurance organization when the policyholder experiences insurance covered loss (Nimo et al. 2011). According to Aidoo et al. (2014), almost all the insurance companies in Ghana provide various insurance schemes (e.g. auto insurance, life and health insurance, fire insurance and burglary) other than crop insurance scheme.

The Dormaa West District of the Brong-Ahafo region of Ghana is known to be one of the major cocoa-producing districts in Ghana. The district is generally an agrarian economy which contributes immensely to the food basket of the country. However, cocoa farmers in this district have over the years lost cocoa crops (particularly at the seedling stage) and yields due to spatial and temporal variability of rainfall (erratic rainfall, drought spills), insect pests and disease outbreak, bush fires, low/poor soil fertility, unavailability of farm inputs (fertilizers, pesticides, high-yielding and pest and disease resistant cocoa seedlings/varieties), high and erratic prices of farm input, ageing cocoa farmers, aging cocoa plantations, technological backwardness, illegal chain saw activities, among others. When such situations occur, farmers lose income, which affect other household members such as children dropping out of school as a result of not being able to pay their school fees and poor health care due to inability to afford medical care. The loss of income may also affect farmers’ ability to purchase farm input for the next crop season, hence, worsening the already poor condition of farmers. To reduce the risk of total loss of income/or crops due to these factors, the insurance of cocoa farms by farmers is crucial. Although, many small-scale cocoa farmers in the Dormaa West District in the Brong-Ahafo Region of Ghana rely on cocoa as a source of revenue (livelihood), there is little information on cocoa farmers’ willingness to insure their crop, premium willing to pay and the willingness of insurance companies to provide crop insurance scheme to farmers. This research therefore seeks to investigate (1) cocoa farmers’ willingness to insure their crop and the premium they are willing to pay, (2) evaluate the factors that influence cocoa farmers’ willingness to insure their crop and the premium to pay and (3) assess the willingness of insurance companies to provide crop insurance to cocoa farmers.

## Literature review

### Analytical technique

Review of literature on willingness to pay for agricultural insurance indicates that there are three ways of estimating farmer’s willingness to pay (WTP) for insurance. They are; contingent valuation method, the revealed preference theory or approach and a combination of the use of theory along with microeconomic household variables and market variables to estimate indirectly the appropriate market premium. Among the stated preference methods, the contingent valuation method have been highly recommended in instances where there is no or little market information and has been widely used by many researchers (Vandeveer and Loehman 1994; Sarris et al. 2006; Liu and Zhang 2011; Nakanyike 2014; Taneja et al. 2014). This is because it helps to simulate the concept of choice in a market situation as respondents have the opportunity to accept or reject the product. As a result of the importance of the contingent valuation method, it has been highly utilized in various agriculture related studies where it was used to elicit farmers’ willingness to pay for a service, product or technology. For example, Ulimwengu and Sanyal (2011) adopted the method in analyzing farmers’ willingness to pay for agricultural services and the method was further used by Kwadzo et al. (2013) and Danso-Abbeam et al. (2014). According to Taneja et al. (2014), the contingent valuation method makes use of surveys that are particularly intended for measuring preferences and willingness to pay. Based on its importance, the contingent valuation was used in this study.

Various studies have used either the double-hurdle model or the Heckman’s sample selection model in determining the willingness to pay for insurance (Cragg 1971; Norris and Batie 1987; Gabre-Madhin et al. 2003; Sindi 2008; Wodjao 2008; Yu and Abler 2010, Musah 2013). In this study, the double-hurdle model was adopted based on its advantage over the Heckman’s sample selection model. The Heckman sample selection model assumes that no zero response will be present in the second hurdle of the analysis once the first hurdle is passed whiles the double-hurdle on the other hand recognizes the possibility of zero observations in the second stage (Wodjao 2008). The possibility of zero response is as a result of the fact that the cocoa farmer may refuse to answer due to a lack of knowledge or how complex the questions are perceived to be. In addition, some cocoa farmers may only have partial information concerning their willingness to pay (Yu and Abler 2010). For such a case, it is possible respondents (farmers) cannot give a number representing their WTP but may recognize the fact that they have a positive WTP. Cragg (1971) suggested a double-hurdle model in which adoption behavior consists of two decisions: an adoption decision, which is a binary choice, modelled using a Logit; and a WTP amount decision, which is a truncated regression model. The Double hurdle, is used in a situation where an event may occur or not and when it does, it takes on continuous positive values (Gabre-Madhin et al. 2003). It is assumed that the cocoa farmer is faced with hurdles in decision making process. Hence, the decision to pay is made first followed by the decision on how much to pay for the insurance. The two equations are assumed to be independent.

## Methods

### The study area

The study was carried out in the Dormaa West District located at the western part of the Brong-Ahafo Region of Ghana. It shares boundaries in the north with the Dormaa Central Municipality, in the east with Asunafo North Municipality, in the west with La Cote D’Ivoire and in the south west with Bia East District. It has a population of 47, 678, comprising of 24, 681 (51.8 %) males and 22, 997 females (48.2 %) (Ghana Statistical Services 2014). The highest mean temperature of the District is about 30 °C which occurs between March and April and the lowest about 26.1 °C in August. The District lies in the sub-humid zone (with annual total rainfall of 800–1200 mm) and has a bimodal rainfall regime. The major economic activities in the District include the cultivation of food and cash crops (including cocoa), poultry and livestock farming, oil palm extraction, cassava processing and sand winning. Soils in the District belong to the Bekwai–Nzema compound Associations. The soil types within the study area support cultivation of both cash and domestic food crops, which include cocoa, coffee, oil palm, citrus, cola-nuts, plantain, cassava and maize, among others. The area is well drained with rivers, mostly perennial due to the bimodal rainfall regime in the area. Notable among them are the Bia, Nkasapim and Pamu rivers. These rivers are mostly used as a source of water for the cultivation of vegetables such as tomatoes, pepper and okra during the dry season. There are however, traditional restrictions on the use of the rivers for fishing (Ghana Statistical Service 2014).

### Sampling technique and sample size

A total of 240 cocoa farmers were selected for the study using the multi-stage sampling technique. The Brong-Ahafo Region of Ghana was purposively selected due to the predominance of cocoa production in the region. The Dormaa West District, known to be one of the major cocoa growing districts in the Brong-Ahafo region was randomly selected. Four cocoa growing communities in the district namely; Nkrankwanta, Diabaa, Krakrom and Kwakuanya were randomly sampled and subsequently sixty (60) cocoa farmers were randomly selected from each of the four cocoa growing communities. The snowball sampling technique was used to select five insurance companies from the Sunyani Metropolis the capital of the region since the total number of insurance companies was not known.

### Instrumentation for data collection

A pre-tested semi-structured questionnaire was developed as an instrument for the study. The structure of questions in the data collection instrument was a combination of close-ended, open-ended and partially close-ended questions. The survey was conducted from December, 2014 to March, 2015.

### Theoretical framework

The theory underpinning this study is utility maximization. Thus, for a farmer to make decision on whether or not to adopt a particular technology or innovation, he does not only consider how to maximize profit from that innovation but on how to attain the highest level of utility otherwise referred to as utility maximization (Sadoulet et al. 1996; McConnell et al. 2009). It was observed that farmers have a level of utility they want to meet and therefore make choices based on their level of utility. For instance, given a number of utility levels ‘K’, a farmer will choose a level that conforms to the highest level of utility given his budget. Such discrete choice scenarios are modeled using the random utility theory (Lubungu et al. 2012).

The utility of a farmer is given as *U*_*ij*_, from choosing alternative *j*. A cocoa farmer will choose whether or not to adopt crop insurance depending on the relative utility levels associated with the two choices. Therefore, the probability that alternative *j* will be chosen is given by

1 $$P(yi = j) = p(Uij \ge Utk|X,\phi k = j) = P(\varepsilon tk - \varepsilon ij \le X_{ij}^{{\prime }} \beta j - X_{ij}^{{\prime }} \beta k|X,\phi k \ne j)$$

where *y*_*i*_ is the observed outcome for the *i*th observation. *i* = 1, …, *N* indexes the cocoa farmer, *j* = 1, …, *j* and *k* = 1, …, *k* are the alternatives being considered, *X* is a vector of farmer, farm and institutional characteristics, *β* is a vector of parameters to be estimated and *ε* is the stochastic random error. Even though the difference in the utilities (V_i_) of adoption and non-adoption are unobserved,

2 $${\text{V}}_{\text{i}} ={\text{ U}}_{\text{ij}} - {\text{U}}_{\text{ik}}$$

The decision of a farmer is taken as a binary outcome such that

3 $$Ji \in j = \left\{ {1\,{\rm if}\,V > 0,\quad 0\,\,\,{\rm otherwise}} \right\}$$

It is assumed from this that the cocoa farmer selects the alternative choices of adoption and non-adoption of crop insurance based on the highest level of utility. This implies that if adoption will enhance his/her highest level of utility, then the farmer will go for that option.

### Empirical model

The first equation in the Double-Hurdle relates to the willingness to adopt crop insurance scheme. A probit regression on the willingness to adopt or not is modeled as:

4 $$\begin{aligned} &WTI = 1\quad {\text{if }}WTI > 0 \quad {\text{and}} \quad WTI = 0\quad{\text{if}} \quad WTI \le 0 \hfill \\ &WTI = zi`\alpha + \varepsilon i \hfill \\ \end{aligned}$$

WTI is a dichotomous variable which assumes a value of 1 and 0 otherwise, z is a vector of farmer, farm and institutional characteristics, *α* is a vector of parameters and *ε*_*i*_ is the error term.

The empirical model for cocoa farmer’s willingness to adopt crop insurance is specified for this study as;

5 $$\begin{aligned} WTI &= \beta_{0} + \beta_{{\mathbf{1}}} Age_{{}} + \beta_{2} Gender_{{}} + \beta_{3} Mstatus_{{}} + \beta_{4} Educ_{{}} + \beta_{5} Hsesize_{{}} \hfill \\ &\quad +\beta_{6} Croparea_{{}} + \beta_{7} Cocoainc_{{}} + \beta_{8} Otherinc_{{}} + \, \varepsilon_{i} \hfill \\ \end{aligned}$$

WTI is the probability that an *i*th cocoa farmer is willing to adopt crop insurance. *β*_*i*_ is the coefficients of the explanatory variables. ε_*i*_ is the error term.

The second hurdle which estimates the amount (premium) cocoa farmers are willing to pay is estimated using a regression truncated at zero. It is expressed as;

6 $$\begin{aligned} WTPamt_{i} = WTPamt_{i}^{*} \, if \, WTPamt_{i}^{*} \, > \, 0 \, and \, WTPamt_{i}^{*} \, = \, 0 \, if \, otherwise \hfill \\ WTPamt^{*} = x_{{\mathbf{i}}}^{{\prime }} \beta + u_{{\mathbf{i}}} \hfill \\ \end{aligned}$$

Where WTPamt* is the observed response on how much cocoa farmers are willing to pay for crop insurance. *χ* is the vector of farmer, farm and institutional characteristics, *β* is a vector of parameters and *u*_i_ is the error term which is randomly distributed.

The empirical model of the truncated regression model (tobit model) is specified for this study as;

7 $$\begin{aligned} WTPamt_{i} &= \beta_{0} + \beta_{{\mathbf{1}}} Age_{{}} + \beta_{2} Gender_{{}} + \beta_{3} Mstatus_{{}} + \beta_{4} Educ_{{}} + \beta_{5} Hsesize \hfill \\ &\quad+ \beta_{6} Croparea_{{}} + \beta_{7} Cocoainc_{{}} + \beta_{8} Otherinc_{{}} + \, \varepsilon_{i} \hfill \\ \end{aligned}$$

where WTP amt_i_ is the amount an *i*th cocoa farmer is willing to pay, *β*_*i*_ are parameters to be estimated and ε_i_ is the error term.

### Definition of variables

#### Age of cocoa farmer (Age)

Age of cocoa farmers was measured in years in this study. It is hypothesized that age can negatively influence a farmer’s willingness to insure his farm. Older farmers are less likely to adopt crop insurance than younger farmers. This is due to the fact that older farmers tend to gather experience from farming and stick to primitive ways of production and do not easily adopt newly introduced technology (Baidu-Forson 1999; Langyintuo and Mulugetta 2005).

#### Gender of cocoa farmer (Gender)

Gender was measured as a dummy variable with male farmers = 1 and female farmers = 0. Gender is hypothesized to be positive. This is due to the fact that male farmers are well endowed with resource such as land than their female counterparts, therefore, the higher the probability of male farmers adopting crop insurance and the higher the amount (premium) paid.

#### Marital status (Mstatus)

Marital status was measured as dummy with married farmers = 1 and single farmers = 0. Marital status is hypothesized to be positive due to the fact that married farmers will consider the survival of their family should any uncertainty strikes (Danso-Abbeam et al. 2014). Therefore, the more likely they would be willing to accept crop insurance policy and pay higher amount as premium.

#### Education of cocoa farmer (Educ)

It is assumed that a farmer who has gained formal education can critically analyze and make own decisions between technologies (Enete and Igbokwe 2009; Caleb and Ramatu 2013). Therefore, a farmer who has gained formal education is more likely to adopt crop insurance and pay higher amounts as premium.

#### Household size (Hsesize)

It is hypothesized that household size can positively or negatively influence a farmer to adopt crop insurance scheme. This is due to the fact that a farmer who has large household size may not want to spend his/her income in any other activities but use it to cater for his/her household. Also, a farmer would adopt crop insurance scheme based on the fact that he would not like to take the risk of losing his farm at the expense of his household in case of disaster.

#### Size of cropped area (Croparea)

The size of cropped area was measured in acres. It is hypothesized that the larger the cropped area, the more likely the farmer would adopt crop insurance. This is because, it is difficult to manage a large portion of farm land than a smaller portion and therefore, the more likely the farmer is to face risk like low yield and pest infestation or the impact a farmer would experience in times of perils is greater with large farm size. On the other hand, the premium a farmer will pay would increase as the size of cropped area increases.

#### Cocoa income (Cocoainc)

The cocoa income was measured as the sale of a kilogram of dried cocoa bean in the year under review. It is hypothesized to be positive. The reason being that as the income of the cocoa farmer increase, the higher the probability of purchasing crop insurance scheme and the higher the premium they will be willing to pay.

#### Income from other sources (Otherinc)

It is assumed that income from other sources can increase the likelihood of a cocoa farmer purchasing an insurance scheme. This is based on the fact that the income from other sources adds up to the income from the sale of cocoa, making the income stream of the farmer greater, hence, the higher the probability to afford an insurance scheme and the higher the amount paid as premium.

## Results and discussion

### Demographic characteristics of farmers

The demographic characteristics of farmers are presented in Table 1. A total of 87.5 % of the respondents were males while 12.5 % were females. Majority (63.8 %) of the respondents were aged between 40 and 59 years while 25.8 % were above 60 years. Only 10.4 % of the farmers were between the ages of 20–39 years. The mean age of the cocoa farmers was 52 years and the maximum age was about 83 years. Majority of the cocoa farmers (71.7 %) were married whiles 28.3 % were single. About 81.2 % of the respondents had formal education, mostly middle/senior high school (43.3 %), primary/junior high school education (34.6 %) and tertiary education (3.3 %), with 18.8 % of the farmers having no formal education. As shown in Table 1 about 94.2 % of the farmers had 11 or more years of farming experience in cocoa production. The average number of years’ farmers have been in cocoa farming in the study area was 21.8.

**Table 1:** Demographic characteristics of cocoa farmers

Variable	Description	Percentage (%)
Gender	Male	87.5
	Female	12.5
Age	20–29	2.1
	30–39	8.3
	40–49	32.5
	50–59	31.3
	Above 60	25.8
Marital status	Single	28.3
	Married	71.7
Educational level	No education	18.8
	Primary/JHS	34.6
	Middle/SHS	43.3
	Tertiary	3.3
Years of farming experience in cocoa	5–10	5.8
	11–15	15.7
	16–20	17.8
	Above 20	60.7

### Major cocoa production constraints/risks

Table 2 presents the risks identified by cocoa farmers in the study area. Insect pests and diseases were identified as the major risk by the farmers, followed by high and erratic prices of farm inputs. Erratic rainfall and long drought spills was identified as a risk which they attributed to climate change. Similarly, farmers complained of illegal chain-saw activities which they said sometimes destroy their crops. A few of the farmers also indicated the incidence of bush fires which sometimes destroy their crops. In a surprising turn, ageing of cocoa farmers was mentioned by some farmers as a risk to cocoa production in the study area.

**Table 2:** Production risk faced by cocoa farmers in the study area

Risks	Percentage (%)
Insects pest and diseases	95.8
High and erratic prices of farm input	81.7
Illegal chain saw activities	30
Erratic rainfall and long drought spills	70
Bush fire	8
Unavailability of farm inputs	25
Ageing cocoa farmers	20

Multiple response analysis

According to Dormon et al. (2004), the incidence of insect pests and diseases is a major challenge in cocoa production in Ghana and has resulted in low yields due to inadequate management. Duguma et al. (1998), Lass (2004) and Dormon et al. (2007) estimate losses by insect pests and diseases to be 30 % of global yields of cocoa annually, whereas site-specific losses ranging from 10 to 80 % annually have been reported. In Ghana, the mirids (capsids) and the black pod disease are the most destructive of the number of insect pests and diseases which attack the developing cocoa tree and the development or ripening of cocoa pod. Farmers in the study area identified mirids as the major insect pest. Other insect pests of cocoa mentioned included termites, mealy bugs, ants, mites, shield bugs, stem borers, and pod borers. This confirms the fact that marids remain the insect pest of economic importance in Ghana (Antwi-Agyakwa 2013). The major cocoa disease mentioned by farmers was the black pod followed by the cocoa swollen shoot virus. Mistletoe a parasitic plant and rodents were also mentioned by some farmers to cause decline in cocoa production in the study area. According to the farmers, capsid and black pod attacks were the major causes of pre-harvest losses in their cocoa farms (65 and 45 % of the respondents respectively). On the other hand, 17 % of the respondents reported that rodents were among the pests of cocoa causing pre-harvest losses and this could be attributed to poor maintenance of farms. However, some farmers could not differentiate between damages caused by these insect pest and diseases and as such misinterpreted the damage of one insect pest or disease for the other.

Climate change and variability is another major risk farmers are facing in the study area. According to Laux et al. (2010) and IPCC (2014), the spatial and temporal variability of rainfall which is reflected by recurrent dry spells and floods is the most important factor affecting crop productivity. Cocoa is highly susceptible to drought and the pattern of cropping of cocoa is related to rainfall distribution. Significant correlations between cocoa yield and rainfall over varying intervals prior to harvest have been reported (Anim-Kwapong and Frimpong 2004). Farmers complained of erratic rainfall and long drought spills resulting in serious shocks in seedlings development and poor yields (Longe and Oyekale 2013). Anim-Kwapong and Frimpong (2004) reported that, dry weather results in soil water deficit resulting in high seedling mortality at the establishment phase. They also indicated that during the pod filling stage, the occurrence of short dry spills affects bean size and hence the quality of cocoa beans produced. This affects the farmer’s income as the quality of cocoa bean is considered when pricing the beans. It was also reported that heavy rainfall between August and October prevents cocoa trees from flowering. Climate change is also expected to alter stages and rates of development of cocoa pests and pathogens, modify host resistance and result in changes in the physiology of host-pathogen/pests interaction (Anim-Kwapong and Frimpong 2004; Kimengsi and Tosam 2013). The most likely consequences are shifts in the geographical distribution of host and pathogen/pests, altered crop yields and crop losses which, will impact socio-economic variables such as farm income, livelihood and farm-level decision making (Anim-Kwapong and Frimpong 2004; Kimengsi and Tosam 2013). According to Anim-Kwapong and Frimpong (2004), the black pod disease of cocoa is closely related to climate and is more prevalent in damp areas. Black pod disease accounts for the bulk of annual production losses of cocoa in Ghana and is most destructive in years when the short dry period between July and August is very wet whiles mirids (capsids) which are sucking insects are usually most active and destructive from September to March particularly when moisture deficit is severe. Excessive shade and bushy farms nearby cocoa farms were identified by 75 % of the farmers to be the major causes of mirids attack on their cocoa farms. Additionally, 70 % of the farmers reported excessive rainfall, excessive shading and stagnant water on their cocoa farms as the cause of black pod disease.

The high incidence of cocoa insect pests and diseases, low soil fertility and bad weather/poor climate (erratic rainfall and long drought spills, strong winds, etc.) results in increased use of pesticides and fertilizers, and planting of improved cocoa varieties by farmers in the study area. However, the farmers indicated that the government of Ghana interventions—supply of high-yielding and disease resistant cocoa seedlings, distribution of fertilizers and mass spraying (spraying of cocoa farms with pesticides) were totally inadequate to revive the sector. For instance, the farmers indicated that cocoa farms in the district are to be sprayed four times a year, between July and November under the Government of Ghana mass cocoa spraying Programme with Ghana Cocoa Board (COCOBOD) approved pesticides. However, in the year of the study, only 51.7 % had their farms sprayed once while only 6.9 % of the farmers had their farms sprayed four times. According to Aneani et al. (2012) and Danso-Abbeam et al. (2014), spraying frequency of the ‘mass spraying exercise’ is not adequate and cocoa farmers are expected to do additional spraying. This situation made farmers in the study area to purchase pesticides from the open market which they claim are very expensive, hence, impacting on cocoa production. This finding confirmed the study by Dormon et al. (2004) which reported that most cocoa farmers in Ghana are not able to control insect pests and diseases on their farms due to high cost of pesticides, spraying equipment and labour (sprayers) resulting in poor yield and in some cases total destruction of cocoa trees. Some of the farmers indicated that they received their fertilizer and pesticide supplies from Ghana Cocoa Board late in the previous year of this study.

Majority of the smallholder farmers interviewed were old and ageing (63.8 % were aged 40–59 years) with only 10.4 % of the farmers between the ages of 20–39 years. This has implication on cocoa productivity and production in the future as ones’ health normally declines with age. There is the likelihood of decline in the production of cocoa in the study area if the current trend does not change. The finding of farmers aging in this study epitomise what the government calls the “generation gap” threatening future production in the world’s second-largest cocoa producing country. The labour-intensive nature of farming could be the reason why youth are not interested in it and will rather migrate to the big cities in search for non-existing jobs. The apparent disinterest in farming by the country’s youth is very much a structural issue that has implication for Ghana’s future cocoa production.

Low yields in cocoa production as a result of these risks is a threat to the livelihood of smallholder farmers, thereby worsening unemployment and poverty as well as the foreign income earnings of the country. Most of these farmers rely on the income from the sale of cocoa to pay their children’s school fees, medical bills and living expenses in general. Crop failure results in children dropping out of school and poor health and nutrition of the family as a whole. The need for farmers to guard against risk of crop failure through farm insurance scheme is therefore crucial.

### Type of insurance schemes used by cocoa farmers

**Fig. 1:**
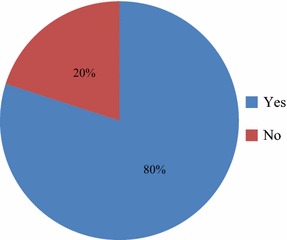
Percentage of cocoa farmers who use insurance scheme in the study area

**Fig. 2:**
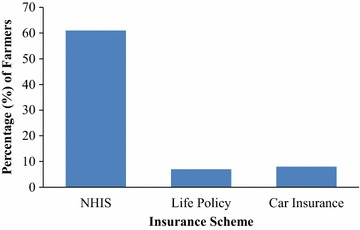
Type of insurance schemes used by cocoa farmers

The study revealed that cocoa farmers have never insured their farms before. In spite of this, some farmers were aware and have used a type of insurance before. Out of the 240 farmers sampled, 80 % have already used a type of insurance scheme whiles 20 % have never used insurance schemes before. Majority of the cocoa farmers (61 %) are using the National Health Insurance Scheme (NHIS), followed by auto insurance (8 %) and life policies (7 %). The reasons farmers gave for using the type of insurance scheme were; to subsidize their medical expenses in times of sicknesses, risk management tool, protection of their properties including family members and protection of their vehicles against future uncertainties (Figs. 1, 2).

### Cocoa farmers’ knowledge and source of information on crop insurance schemes

Table 3 presents summary of cocoa farmers’ knowledge and sources of information on crop insurance. From the Table, 40 % of the farmers were aware or had knowledge of crop insurance scheme whiles 60 % had no knowledge of crop insurance scheme. Thirty percent (30 %) out of the 40 % who had knowledge of crop insurance schemes indicated that it served as a form of compensation during uncertainties whiles the rest (10 %) indicated that crop insurance scheme was a type of support from government. The cocoa farmers had information on crop insurance schemes through radio (25 %), Agricultural extension agents (AEA) (10 %) and Farm Based Organization (FBO) (5 %).

**Table 3:** Cocoa farmer’s knowledge and sources of information on crop insurance

	Frequency	Percentage
Knowledge of crop insurance scheme		
Yes	96	40.0
No	144	60.0
Sources of information on crop insurance scheme		
Media	60	25
Agricultural extension agents (AEA)	24	10
Farm Based Organization (FBO)	12	5

### Farmers’ willingness to insure and pay a premium for crop insurance

Cocoa farmers’ willingness to insure and willingness to pay an amount for crop insurance per annum are presented in Table 4. From the table, majority (85.4 %) of the cocoa farmers were willing to insure their cocoa farms whiles 14.6 % were not willing to insure their cocoa farms. This indicates farmers’ awareness of the importance of crop insurance. The farmers indicated that the crop insurance could guard against loss of crops through theft and perils (fire outbreak, flood and drought). The maximum amount cocoa farmers were willing to pay as premium per acre per annum in order to insure their cocoa farms was GH¢128.40, the minimum amount was GH¢32.10 and the average amount was GH¢49.32. Majority (67.80 %) of the cocoa farmers were willing to pay 10 % of their total production cost (i.e. GH¢ 321.0 on the average) per acre as insurance premium while only 6.83 % were willing to pay 40 % of their production cost as premium. The farmers who were not willing to pay a premium to insure their cocoa farms indicated lack of information or knowledge on crop insurance scheme.

**Table 4:** Cocoa farmers’ willingness to insure and the amount to pay for crop insurance

Variable	Description	Percentage (%)
Willing to insure cocoa farms	Yes	85.4
	No	14.6
Percentage of total production cost/acre/annum farmers are willing to pay as premium	10	67.80
	20	17.56
	30	7.80
	40	6.83
Minimum premium willing to pay/acre/annum	GH¢32.10	67.80
Maximum premium willing to pay/acre/annum	GH¢128.40	40
Average amount farmers are willing to pay per acre per annum	GH¢ 49.32	

### Factors influencing farmers’ willingness to insure and willingness to pay for crop insurance

The factors influencing farmers’ willingness to insure and willingness to pay amount for crop insurance are presented in Table 5. From the Table, gender has no significant influence on both willingness to insure and willingness to pay for crop insurance. This result is consistent with the findings of Ćurak et al. (2013) who assert that gender has no significant relationship with insurance policy. Even though it was not significant, it did not conform to the a-prior expectation which is positive. This may be as a result of the fact that even though male farmers are well endowed, they are risk loving compared to female farmers, hence, may not see the need to insure their farms and pay for insurance premium. Studies have shown that women are more risk averse than men (Palsson 1996; Donkers et al. 2001; Hartog et al. 2002; Cohen and Einav 2007; Dohem et al. 2011). This means that men are risk loving and would be less likely to take risk reduction strategies such as insurance even though they are endowed than women.

**Table 5:** Double Hurdle Model on factors influencing farmers’ willingness to insure and pay for crop insurance

Willingness to insure					Willingness to pay		
Probit					Tobit		
Variables	dy/dx	Coeff.	Std. Error	*p* > z	Coeff.	Std. Error	*p* > z
Gender	−0.00668	−0.14677	0.40268	0.716	−4.05602	3.19595	0.206
Age	0.002997	0.06020	0.02545	0.018**	1.42499	0.141128	0.000***
Mstatus	0.23526	1.86291	0.38959	0.000***	−27.7962	3.00312	0.000***
Hsesize	−0.01774	−0.35628	0.10474	0.001***	2.46747	0.68126	0.000***
Educ	0.03674	2.11833	0.72420	0.003***	−2.98324	4.13594	0.472
Croparea	−0.00277	−0.05572	0.01881	0.003***	0.56288	0.16897	0.001***
Cocoainc	0.01446	0.29049	0.61013	0.634	−8.53390	4.84956	0.080*
Otherinc	−0.00244	−0.04898	0.36340	0.893	−0.00444	2.41869	0.999
Constant	–	−2.78862	6.47976	0.667	54.48673	48.37018	0.261

Regression diagnostics	Value	Regression diagnostics	Value
Number of observations	240	Cragg & Uhler’s R^2^	0.690
Log-likelihood	−40.4804	Efron’s R^2^	0.545
LR χ^2^ (8)	118.437	McKelvey and Zavoina’s R^2^	0.704
Prob > χ^2^	0.000	Count R^2^	0.917
Pseudo R^2^	0.5940	AIC	0.412
Maximum likelihood R^2^	0.390	Variance of y*	3.375
McFadden’s R^2^	0.594	Variance of error	1.00

***, **, and * 1, 5 and  10 % significant levels respectively

However, the age of farmers was statistically significant at 5 % and positively influenced willingness to insure crops. This however did not conform to the a prior expectation. The result revealed that as a farmer’s age increases, he/she is more likely to insure his/her cocoa farm. The result however contradicted findings by Baidu-Forson (1999), Langyintuo and Mulugetta (2005) and Aidoo et al. (2014) which stated that older farmers are less likely to insure their farms. Again, age positively influenced the amount a cocoa farmer is willing to pay for crop insurance and was statistically significant at 1 %. This shows that as the age of a cocoa farmer increases by 1 year, the amount he/she is willing to pay increases by GH¢1.42.

Similarly, marital status was statistically significant at 1 % and positively influenced farmers’ willingness to insure their cocoa farms. The result again revealed that the likelihood of a married farmer insuring his/her farm is 0.235. However, marital status negatively influenced the amount (premium) cocoa farmers are willing to pay for crop insurance and this was statistically significant at 1 %. Thus, the amount (premium) a farmer was willing to pay if married decreases by GH¢27.80.

Household size was statistically significant at 1 % and negatively influenced a farmer’s willingness to insure his/her cocoa farm. This means that as the household size of a cocoa farmer increases by one person, the likelihood of him/her insuring his/her farm reduce by 0.018. This result is in line with findings of Falola et al. (2013) and Danso-Abbeam et al. (2014). However, the amount a farmer is willing to pay increases by GH¢2.47 as his/her household size increase by one person.

The educational level of cocoa farmers positively influenced their willingness to insure their cocoa farms and was significant at 1 %. Thus, the higher the educational level of a farmer, the more likely he/she would be willing to insure his/her cocoa farm. This conformed to the a prior expectation and is in line with findings of Piyasiri and Ariyawardana (2002), Falola et al. (2013), Aidoo et al. (2014) and Danso-Abbeam et al. (2014). The results could be explained by the fact that a farmer who gains formal education can critically analyze and make own decisions between technologies as revealed by Enete and Igbokwe (2009) and Caleb and Ramatu (2013). However, education did not significantly influence the premium a cocoa farmer was willing to pay for crop insurance.

Size of cropped area was statistically significant at 1 % and negatively influenced a farmer’s willingness to insure his/her cocoa farm. Thus, the bigger the cropped area the less likely a farmer would be willing to insure his/her farm. From the results, as the size of cropped area increases by one acre, the probability that a cocoa farmer would insure his farm reduces by 0.003. The results again revealed that the amount (premium) farmers are willing to pay for crop insurance is positively influenced by size of cropped area and statistically significant at 1 %. This means that as size of cropped area increases by one acre, the amount (premium) farmers are willing to pay for crop insurance increases by GH¢0.56. This contradicts the findings of Kumar et al. (2011) which reported that size of cropped area negatively influence willingness to pay an amount for crop insurance.

Income from cocoa did not significantly influence a farmer’s willingness to insure his/her cocoa farm. However, it had a positive effect and conforms to the a-prior expectation. This findings contradicts the findings of Danso-Abbeam et al. (2014) who reported that cocoa income significantly and positively influence a farmer’s willingness to insure his/her cocoa farm. However, cocoa income was statistically significant at 10 % and negatively influenced the premium cocoa farmers are willing to pay for crop insurance. Thus, if a cocoa farmer’s income increases by GH¢1.00, the amount he/she would be willing to pay decreases by GH¢8.53.

Income from other sources was not significant and negatively influenced both farmer’s willingness to insure his/her cocoa farm and the amount a cocoa farmer would be willing to pay for crop insurance. This could be explained by the fact that as a farmer diversifies his income sources, he/she feels secured, therefore, would not be willing to insure and pay for crop insurance.

### Insurance companies’ willingness to provide crop insurance scheme to cocoa farmers

The insurance companies interviewed provide insurance schemes other than agricultural insurance scheme. The insurance policies offered by these companies were auto insurance (comprehensive and third party auto insurance), fire and related risks (loss of property through fire, explosion, and lighting), burglary (loss of property through theft) and life (injuries and death).

It was noted that all the insurance companies did not have crop insurance scheme as part of their operations. However, 80 % of the insurance companies were aware of crop insurance schemes. Reasons were sort as to why they had no crop insurance policy. These companies indicated that the high risk of the agricultural sector was the main reason for not offering insurance policy as the sector in Ghana is highly dependent on rainfall with most farmers using low input or no input in their farm. For most of these companies, the smallholder farmers who form the larger portion of farmers may not be able to afford the crop insurance. Some companies however indicated there is a proposal to undertake crop insurance and it is awaiting approval.

Although the insurance companies did not have crop insurance as part of their insurance policies, 40 % of the companies were willing to carry out crop insurance for cocoa farmers if proposal is approved. The insurance companies indicated that they are willing to provide crop insurance for cocoa farms because cocoa is an export commodity and therefore, generate enough income for farmers. In addition, they indicated that cocoa is a tree crop and therefore, farmers cannot easily switch to different crop once they log on to the crop insurance policy (Unlike annual crops where a farmer can decide not to grow that same crop the following year which can distort continues flow of payment and record). The 60 % of the companies who were reluctant to undertake crop insurance gave the following reasons; agriculture is highly risky, lack of income diversification activities among the farmers, agricultural produce are highly perishable and lastly, farmers do not use modern ways of cultivation (lack of adoption of technology among farmers).

Even though the insurance companies were willing to provide crop insurance to cocoa farmers, the insurance companies indicated the need for awareness creation and education of farmers on the need to adopt crop insurance schemes, adopt modern ways of cultivation and good record keeping (Table 6).

**Table 6:** Awareness of insurance companies on farm insurance policies and interest in providing farm insurance policies or schemes to farmers

Variable	Description	Percentage (%)
Awareness on crop insurance	Yes	80
	No	20
Interest in crop insurance polices	Yes	40
	No	60

## Conclusions and recommendations

The study shows that majority (80 %) of cocoa farmers have used National Health Insurance Scheme (NHIS), life policy and auto insurance but have never used crop insurance. This is due to lack of crop insurance scheme. However, 40 % of the farmers are aware or have knowledge of crop insurance from the media, AEAs and FBOs. This shows that crop insurance is not popular among cocoa farmers.

Majority of the cocoa farmers (85.40 %) were willing to insure their cocoa farms. However, only 6.83 % of the farmers are willing to pay the maximum premium of GH¢128.40. It could be deduced that though the farmers were willing to insure their farms, their willingness to insure did not mean majority would pay higher premium.

In assessing factors influencing farmers’ willingness to insure and willingness to pay for crop insurance, the double-hurdle model was used. Age, marital status and education significantly and positively influenced cocoa farmers’ willingness to insure their farms whiles household size and cropped area significantly and negatively influenced cocoa farmer’s willingness to insure their farms. Similarly, age, household size and cropped area significantly and positively influenced the premium cocoa farmers were willing to pay whiles marital status and cocoa income significantly and negatively influenced the premium cocoa farmers were willing to pay.

Insurance companies did not have crop insurance scheme as part of their operations although majority were aware of crop insurance scheme. However, the insurance companies were willing to provide crop insurance to cocoa farmers if only farmers adopt modern ways of cultivation to reduce the risk involved in production and also have good record keeping.

It is recommended that cocoa farmers be well informed and educated on crop insurance and the need to insure their cocoa farms since majority were not aware or had no knowledge of crop insurance. This could also increase the premium they will be willing to pay for crop insurance. Farmers should be involved in planning the crop insurance scheme in order to conclude on the premium to be paid by the cocoa farmers. There is a need for further research to determine the premium farmers are willing to pay and the factors which influence the premium farmers are willing to pay.

## Abbreviations

GH: Ghana
WTP: willingness to pay
WTI: willingness to insure
GDP: gross domestic product
NHIS: National Health Insurance Scheme
AEA: agricultural extension agents
FBO: farm based organization
COCOBOD: Ghana Cocoa Board
